# Autophagy induces G0/G1 arrest and apoptosis in menstrual blood-derived endometrial stem cells via GSK3-β/β-catenin pathway

**DOI:** 10.1186/s13287-018-1073-0

**Published:** 2018-11-28

**Authors:** Jiang Du, Xinxing Zhu, Rui Guo, Zhihao Xu, Fang Fang Cheng, Qing Liu, Fen Yang, Lihong Guan, Yanli Liu, Juntang Lin

**Affiliations:** 1 College of Biomedical Engineering, Xinxiang Medical University, Xinxiang, 453003 China; 2 Stem Cell and Biotherapy Engineering Research Center of Henan, Xinxiang Medical University, East of JinSui Road #601, Xinxiang, 453003 Henan China; 3 College of Life Science and Technology, Xinxiang Medical University, Xinxiang, 453003 China; 4 Henan Key Lab of Biological Psyshiatry, The Second Affiliated Hospital of Xinxiang Medical University, Xinxiang, 453003 China

**Keywords:** Autophagy, Menstrual blood-derived endometrial stem cells, G0/G1 arrest, Gsk3β, Apoptosis

## Abstract

**Background/aims:**

Menstrual blood-derived endometrial stem cells (MenSCs) emerge as an ideal source for cell-based treatment in regenerative medicine and immunotherapy. However, the major obstacle is the low survival rate in tissues and the limited expansion number. Autophagy is an intracellular metabolic self-degradative process which plays important roles in normal cellular division and survival, and the present study aimed to explore the related mechanisms between autophagy and survival of MenSCs in vitro and in vivo.

**Methods:**

The MenSCs were obtained from menstrual blood procured from healthy female donors. In vitro, MenSCs were exposed to rapamycin and Earle’s balanced salts solution (EBSS). We evaluated the MenSCs immunophenotypic cell cycle distribution by propidium iodide (PI) staining and cell apoptosis by Annexin V/PI staining as well as their proliferative potential by the MTT assay. We also assessed the expression of genes associated with the cell cycle and Gsk3β signaling pathway by western blot analysis. We depressed Atg5 and Gsk3β expression by short hairpin RNA (shRNA) and undertook the experiments. Moreover, the labeled MenSCs were observed and counted with DiI after transplantation into the mice via the tail vein by microscopy in vivo.

**Results:**

In vitro, rapamycin and starvation induced autophagy of MenSCs. Hyperactive autophagy significantly induced G0/G1 arrest and slightly promoted apoptosis of MenSCs. Meanwhile, autophagy could stimulate p-GSK3β expression in MenSCs. Further, knockdown GSK3β can accelerate the proliferation of MenSCs by shRNA and CHIR99021. Moreover, the shGSK3β MenSCs showed strong proliferative activity in vitro and in vivo.

**Conclusions:**

Our results indicate that autophagy induced G0/G1 arrest and apoptosis of MenSCs via GSK3β/β-catenin pathway. Inhibiting autophagy or reduced GSK3β levels may improve survival rate in vivo, thus playing roles in MenSCs therapy.

## Background

Mesenchymal stem cells (MSCs) constitute a heterogeneous subset of stromal regenerative cells which can be harvested from several adult tissues. Menstrual blood-derived endometrial stem cells (MenSCs) are substantially MSCs that can be obtained in a non-invasive manner [1, 2]. They retain expression of the MSC markers CD29, CD73, CD90, and CD105 and can be induced into multiple cell lineages under certain conditions [3]. Due to their rapid proliferation rate, low immunogenicity, and low tumorigenicity, MenSCs are used extensively in cell-based therapeutics and regenerative medicine [4]. Multiple studies have described the beneficial effects of MSCs for ameliorating various diseases, including inflammatory disorders, diabetes, and metabolic diseases [5, 6]. However, the primary disadvantage in stem cell therapy is the low survival rate at the disease site [7]. Therefore, it is significantly important to improve the stem cell survival and proliferation in vivo for stem cell therapy.

Autophagy is an evolutionarily conserved process that degrades cellular components by lysosomes [8]. This dynamic process, defined as autophagic flux, includes autophagosome synthesis and maturation, autophagosome-lysosome fusion, and cargo degradation [9]. The autophagy pathway is positively regulated by starvation, mTOR signaling inhibition, and immune-related signaling molecules and negatively regulated by Atg5, Atg7, and Beclin1, which are essential for autophagosome formation in many cell types [10, 11]. Emerging evidence indicates that autophagy plays a consistent role in the modulation of cell proliferation and apoptosis in a wide variety of cell types, including MSCs [12, 13]. For instance, autophagy regulates the apoptosis of bone marrow-derived MSCs (BMMSCs) under hypoxic condition via the AMPK/mTOR pathway [14]. Although MSCs isolated from different sources share many biological characteristics, they show differences in their mechanisms of cell proliferation and apoptosis [15–17]. It is necessary to understand the mechanism of how autophagy effects on replication and apoptosis of MenSCs, thus playing an important role for stem cell therapy using MenSCs.

In this study, we demonstrated the effect of autophagy in MenSCs on their proliferation and apoptosis. Overactivation of autophagy using starvation and rapamycin induced G0/G1 arrest and apoptosis of MenSCs. However, the abnormal division and apoptosis of MenSCs can be rescued by downregulation of GSK3β. Further, inhibition of GSK3β improved the cell survival of MenSCs after transplantation. Our findings identify autophagy to be a critical regulator for improving the survival rate of MenSCs during their treatment for diseases.

## Materials and methods

### Animals

Male BALB/c mice (6 to 8 weeks old) were purchased from Vital River Laboratories (Beijing, China) and kept under specific pathogen-free conditions on a 12-h light-dark cycle. Guidelines of the Animal Care Committee of Xinxiang Medical University were followed when carrying out in vivo experiments.

### Isolation and culture of MenSCs

The menstrual blood samples isolated from healthy female donors and the samples were mixed with equal volume of PBS containing 0.25 mg/ml amphotericin B, 100 U/ml penicillin, 100 mg/ml streptomycin, and 2 mM EDTA under sterile conditions. Shortly thereafter, the menstrual blood samples were delivered into a laboratory and subjected to standard Ficoll (GE, Cat#17-1440-02, USA) procedures within 72 h. After centrifugation, the mononuclear cell layer was carefully transferred to a new tube and washed twice with × 1 PBS. Then, the cells were resuspended and cultured in high-glucose DMEM medium (HyClone, Cat#SH30022.01, USA) supplemented with 10% FBS (Gibco, Cat#10099-141, USA), 100 U/ml penicillin, and 100 mg/ml streptomycin. The culture medium was changed on the third day to remove non-adherent cells. When the cells reaching 80–90% confluence (P0), the cells were passaged using 0.25% Trypsin-EDTA. The third passage cells were used for subsequent experiments.

### Cell viability assay

The MTT assay was used to measure MenSCs viability. MenSCs (1000/well) were seeded in 96-well plates overnight. To detect the effects of rapamycin on MenSCs viability, cells were incubated with different concentrations (1, 10, 50, and 300 nM and 1 μM) of rapamycin for 24 h and different time with 50 nM or 1 μM rapamycin (Selleck, Cat#S1039, USA) for 2, 4, 6, and 8 days; normal culture media with a proportionate DMSO were used for the control group.

To detect the beneficial effects of CHIR99021 (Selleck, Cat#S1263, USA) on MenSCs treated with rapamycin, cells were first co-cultured with CHIR99021 (1 μM) and different concentrations (1, 10, 50, and 300 nM and 1 μM) of rapamycin for 24 h; normal culture media with a proportionate DMSO were used for the control group.

To detect the proliferation rates of shGFP, shATG5, and shGSK3β MenSCs, cells (1000/well) were seeded in 96-well plates overnight. All the groups are cultured with normal culture media.

At the prespecified time points, 10 μl of MTT solution (Songon, Cat#A600799, China) was added to the cells. After incubation for another 4 h and DMSO for 5 min, the optical density (OD) values were determined at 490 nm using a microplate reader. Each group was tested in triplicate for five replicate wells.

### Generation of ShATG5 and ShGSK3β stable MenSCs

Cells were infected with lentivirus particles in 6-well plates. The medium was changed a day prior to lentivirus infection. MenSCs were infected with vector lentivirus particles expressing ShATG5, ShGSK3β, or shGFP as non-targeting control particles. Lentivirus particles were mixed in 1 ml Lipofectamine 2000 (Thermo, Cat#11668027, USA) and added along with 1 μl Polybrene (Santa Cruz, Cat#sc-134220, USA). MenSCs were then incubated for 16 h, following which medium was changed to complete DMEM. After 3 days following lentivirus infection, MenSCs were selected and cultured by treatment with 2 μg/ml puromycin treatment.

### Cell cycle assay

MenSCs were seeded at 2 × 105 cells/dish in 100 mm cell culture dishes. At 12 h after seeding, the cells were washed with 10 ml PBS three times, and then, rapamycin (50 nM or 1 μM), rapamycin/CHIR99021 (50 nM/1 μM or 1 nM/1 μM), or control medium was added respectively. After 24 h, cells were harvested and fixed in ice-cold 70% ethanol for 24 h. Then, the cells were incubated in 10 μg/ml propidium iodide solution (Sigma, Cat#P4864, USA) containing 200 μg/ml RNase A. Beckman FACS was used for the test.

### Cell apoptosis assay

MenSCs were exposed with rapamycin (50 nM), Earle’s balanced salts solution (EBSS) 3 h, rapamycin/CHIR99021 (50 nM/1 μM), EBSS 3 h/CHIR99021 1 μM, or control media at 37 °C for 48 h, and apoptotic cells were detected using FITC-Annexin V and propidium iodide (Beyotime, Cat#C1063, China). Briefly, after washing with cold PBS, cells were resuspended in 190 μl binding buffer with 10 μl FITC-Annexin V and 5 μl propidium iodide and then incubated for 15 min at room temperature. Numbers of apoptotic cells were determined by flow cytometry.

### Western blot analysis

To detect autophagy marker expression, the following antibodies were used: LC3B (CST, Cat#3638, USA), p62 (CST, Cat#8025, USA), and Beclin-1 (Abcam, Ca#ab55878, USA); to detect the expression levels of cell cycle-related proteins, the following antibodies were used: Cdk2 (CST, Cat#2546, USA) and Cdk4 (CST, Cat#12790, USA); to detect the expression levels of GSK3β/β-catenin signaling-related proteins, the following antibodies were used: GSK3β(CST, Cat#12456, USA), p-GSK3β (CST, Cat#5558, USA), and β-catenin (CST, Cat#8480, USA). Rabbit monoclonal β-tubulin (Boster, Cat#BM4264, China) and GAPDH (Boster, Cat#BM3874, China) antibodies were used throughout as a loading control.

Cells were harvested in RIPA lysis buffer with a protease inhibitor, and cell lysates (15 μg) were electrophoresed through polyacrylamide gels. After transferring the proteins to PVDF membranes, the membranes were blocked with 5% non-fat milk in PBS for 1 h and then incubated separately with primary antibodies at 4 °C overnight, followed by incubation with HRP-conjugated secondary antibodies (Boster, Cat#BM2002, China). Antibodies were detected using enhanced chemiluminescence reagent.

### RNA extraction and real-time PCR analysis

MenSCs were exposed with rapamycin (50 nM, 1 μM), EBSS (3 h, 6 h), rapamycin/CHIR99021 (50 nM/1 μM, 1 μM/1 μM), EBSS 3 h/CHIR99021 1 μM, EBSS 6 h/CHIR99021 1 μM or control media at 37 °C for 24 h. Then, the cells were washed and harvested, and total RNA was extracted with TRIzol Reagent according to the manufacturer’s instructions and reverse transcribed into cDNA using the PrimerScript RT reagent Kit (Takara, Cat#RR047A, Japan). All the real-time PCR reactions were carried out using the SYBR Premix Ex Taq reagent kit (Takara, Cat#RR420A, Japan) and an ABI 7500 real-time PCR system. The reaction was carried out according to the manufacturer’s instructions in triplicate. mRNA expression was quantified using the delta-delta CT method, and GAPDH served as the internal control. The following primers were used: P16—forward 5′-AACGCACCGAATAGTTACGG-3′ and reverse 5′-CACCAGCGTGTCCCAGGAAG-3′; P21—forward 5′-TGTGATGCGCTAATGGCG-3′ and reverse 5′-AAGTCGAAGTTCCATCGCTCA-3′; GAPDH—forward 5′-GCACCGTCAAGGCTGAGAAC-3′ and reverse 5′-ATGGTGGTGAAGACGCCAGT-3′.

### Cell labeling and imaging

The cell labeling procedures were performed according to the protocols of the manufacturers. In brief, MenSCs were incubated at the concentration of 2.5 μg/ml fluorescent lipophilic tracer DiI (Sigma, Cat#468495, USA) in growth medium for 12 h at 37 °C. Then, the cells were detached and washed twice with PBS. For transplantation, 2 × 10^5^ DiI-labeled cells (0.2 ml) were injected into BALB/c mice from the tail vein (*n* = 3); the mice that received 10 μg DiI in 0.2 ml PBS were taken as controls.

One week after MenSCs transplantation, all the mice were killed. The heart, liver, lung, spleen, and kidney were fixed in 4% formaldehyde solution overnight and then dehydrated in 30% sucrose solution overnight. Subsequently, the specimen was embedded in OCT compound, frozen in liquid nitrogen, and stored at − 80 °C. Finally, the samples were adjacently sectioned with 20-μm thickness on the poly-l-lysine-coated slides with a cryotome and imaged under a fluorescence microscope.

### Statistical analysis

All experiments were repeated at least three times independently. Statistical analyses were performed using GraphPad Prism version 6 (GraphPad Software). A pairwise comparison was performed by two-tailed unpaired Student’s *t* test. Statistical comparisons between groups were performed using one-way ANOVAs followed by the Tukey test or Dunnett’s test. Differences were considered significant at the level *p* < 0.05. *p* values < 0.05 were considered significant, *p < 0.05, **p < 0.005, ***p < 0.001. Data are presented as an arithmetic mean ± standard error of the mean (SEM).

## Results

### Isolation and identification of MenSCs

**Fig. 1:**
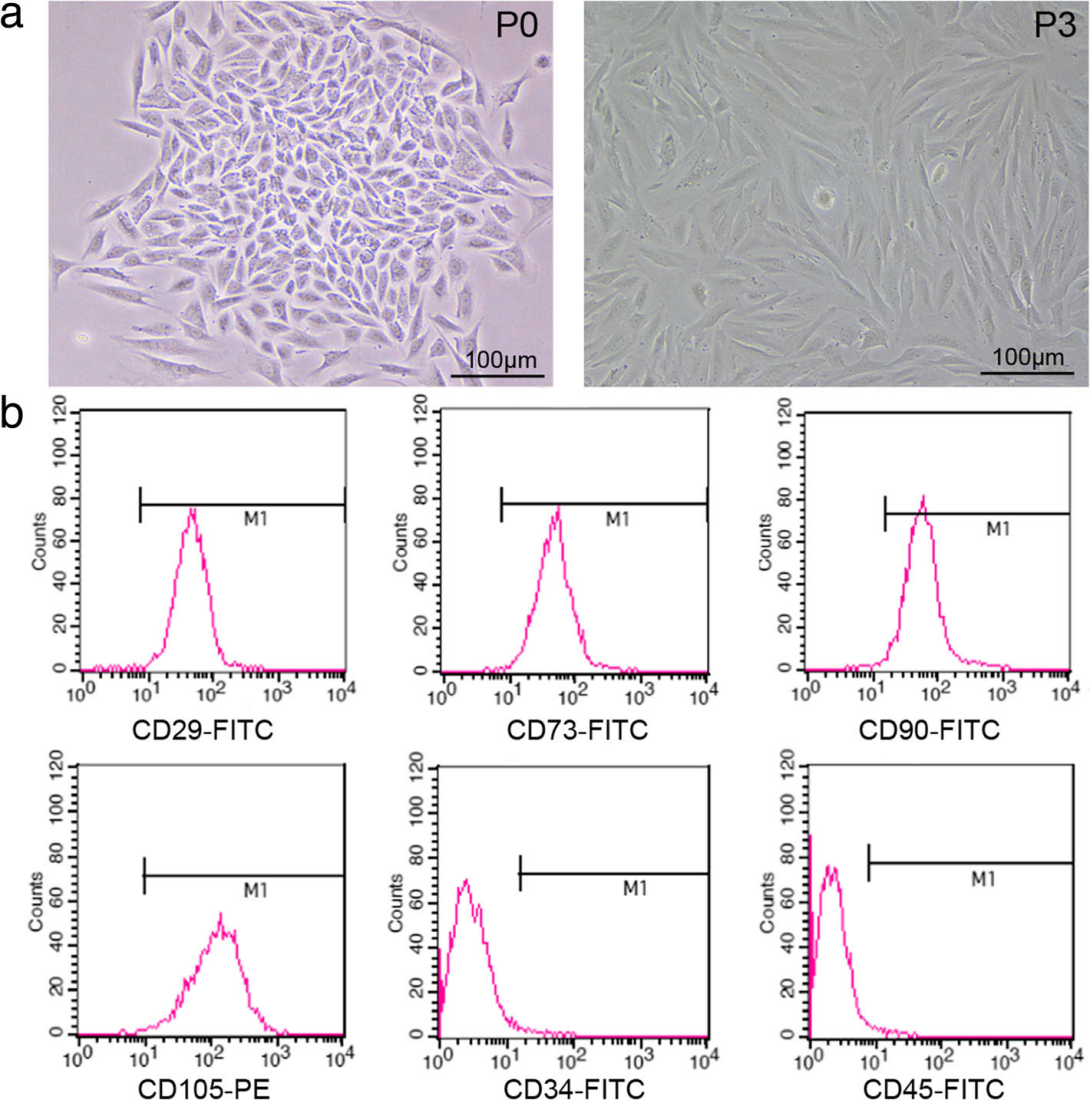
Morphology and immunophenotypes of MenSCs. **a** Morphologic images of MenSC-formed clones (P0 and P3) photographed under a microscope. **b** Cells of P3 express high levels of CD29, CD73, CD90, and CD105. However, they failed to express CD34 and CD45. Scale bars, 100 μm

MenSCs were purified from the menstrual blood samples through density centrifugation using a Ficoll gradient. After that, a colony-like morphology was clearly observed in the primary cultures of MenSCs. The MenSCs grew continuously and expanded to form confluent cultures of adherent cells with a fibroblastic morphology (Fig. 1a). The phenotypic profile of the MenSCs was determined by flow cytometry analysis. The MenSCs were positive expression of mesenchymal stem cell markers CD29, CD73, CD90, and CD105 and negative expression of hematopoietic cell marker CD45 or CD34 (Fig. 1b).

### Rapamycin and starvation induce autophagy of MenSCs

**Fig. 2:**
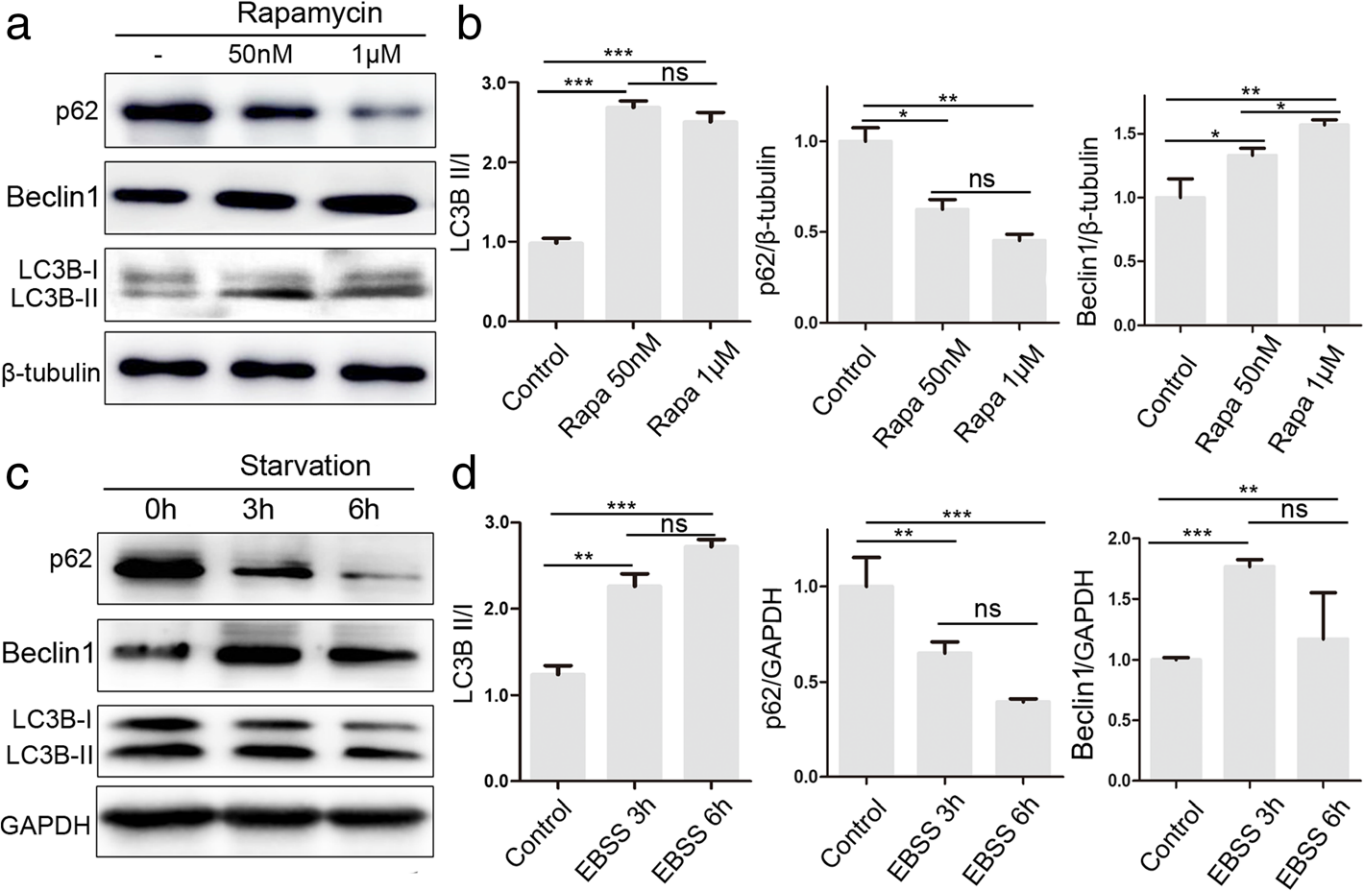
Rapamycin and starvation induce autophagy of MenSCs. **a**, **b** WB was used to detect rapamycin-induced (50 nM, 1 μM) changes in autophagy proteins, LC3, Beclin1, and p62. Significant differences were detected by one-way ANOVA followed by Tukey test; ns represents not significant, *p < 0.05, **p < 0.01, ***p < 0.001. **c**, **d** WB was used to detect starvation-induced (EBSS 3 h, 6 h) changes in autophagy proteins, LC3, Beclin1, and p62. One-way ANOVA followed by Tukey test; ns represents not significant, *p < 0.05, **p < 0.01, and ***p < 0.001. Data are provided as means ± SEM

Previous studies revealed that autophagy is involved in the molecular biology of MSCs. We investigated the autophagy in MenSCs pretreated with starvation or rapamycin, a well-described inducer of autophagy. Western blot analysis showed that the ratio of LC3 I/II and Beclin1/β-tubulin decreased but that the ratio of p62/β-tubulin increased after treatment with rapamycin 50 nM or 1 μM for 24 h (Fig. 2a, b). Meanwhile, we obtained the same results of LC3 I/II, Beclin1, and p62 expression pattern after starvation for 3 or 6 h (Fig. 2c, d). These results suggested that starvation or rapamycin induces autophagy in MenSCs.

### Autophagy induces G0/G1 arrest and inhibits proliferation of MenSCs

**Fig. 3:**
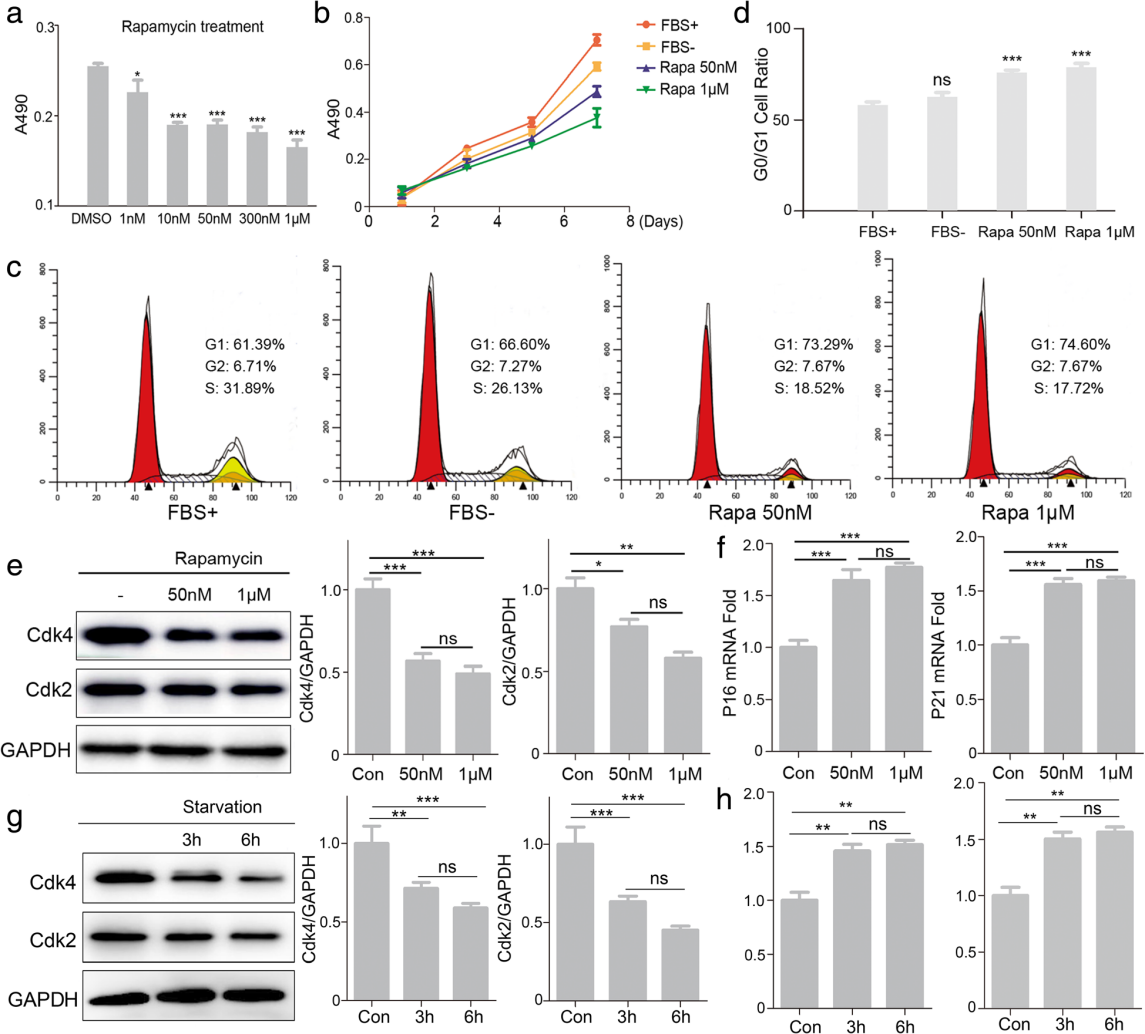
Autophagy induces G0/G1 arrest and inhibits proliferation of MenSCs. **a**, **b** Cell proliferation with rapamycin at different concentration or time was assessed using MTT assay. Significant differences were detected by one-way ANOVA followed by Dunnett’s test, *p < 0.05 and ***p < 0.001, versus DMSO. **c**, **d** Flow cytometric analysis and of cell cycle distribution, one-way ANOVA followed by Dunnett’s test; ns represents not significant, ***p < 0.001, versus FBS+. **e** WB was used to detect Cdk2 and Cdk4 expression in MenSCs treated with rapamycin, one-way ANOVA followed by Tukey test; ns represents not significant, *p < 0.05, **p < 0.01, and ***p < 0.001. **f** Real-time PCR analysis of P21 and P16 mRNA expression in MenSCs treated with rapamycin, one-way ANOVA followed by Tukey test; ns represents not significant, ***p < 0.001. **g** WB was used to detect Cdk2 and Cdk4 expression in MenSCs treated with starvation, one-way ANOVA followed by Tukey test; ns represents not significant, **p < 0.01 and ***p < 0.001. **h** Real-time PCR analysis of P21 and P16 mRNA expression in MenSCs treated with starvation. Statistical analysis is based on one-way ANOVA followed by Tukey test; ns represents not significant, **p < 0.01. All data are provided as means ± SEM

To examine whether autophagy effected on the proliferation of MenSCs, MTT assay was carried out on MenSCs treated with gradient concentrations of rapamycin. The proliferation rates of MenSCs were significantly decreased when treated with 10, 50, and 300 nM and 1 μM rapamycin for 24 h (Fig. 3a). Then, MenSCs were exposed to 50 nM and 1 μM rapamycin for 2, 4, 6, and 8 days (Fig. 3b). The ratio of proliferation rate was obviously inhibited when using rapamycin treatment. Furthermore, propidium iodide staining was used to determine cell cycle. It shows that rapamycin significantly altered the cell cycle distribution of MenSCs with a tendency towards an increased percentage of cells in the G0/G1 phase of the cell cycle (Fig. 3c, d). Meanwhile, we activated cell autophagy using rapamycin or starvation, and western blot was used to detect protein expression levels of Cdk2 and Cdk4 (Fig. 3e, g). The results showed that expressions of Cdk2 and Cdk4 were decreased significantly when treated with rapamycin or starvation. We also assessed mRNA expression levels of two the factors that negatively regulate cell cycle P16 and P21. Results of real-time PCR showed that cells treated with rapamycin or starvation significantly have higher mRNA levels of both P16 and P21 compared to the control group (Fig. 3f, h). These results suggest that autophagy inhibited proliferation of MenSCs by a mechanism associated with altered cell cycle progression.

**Fig. 4:**
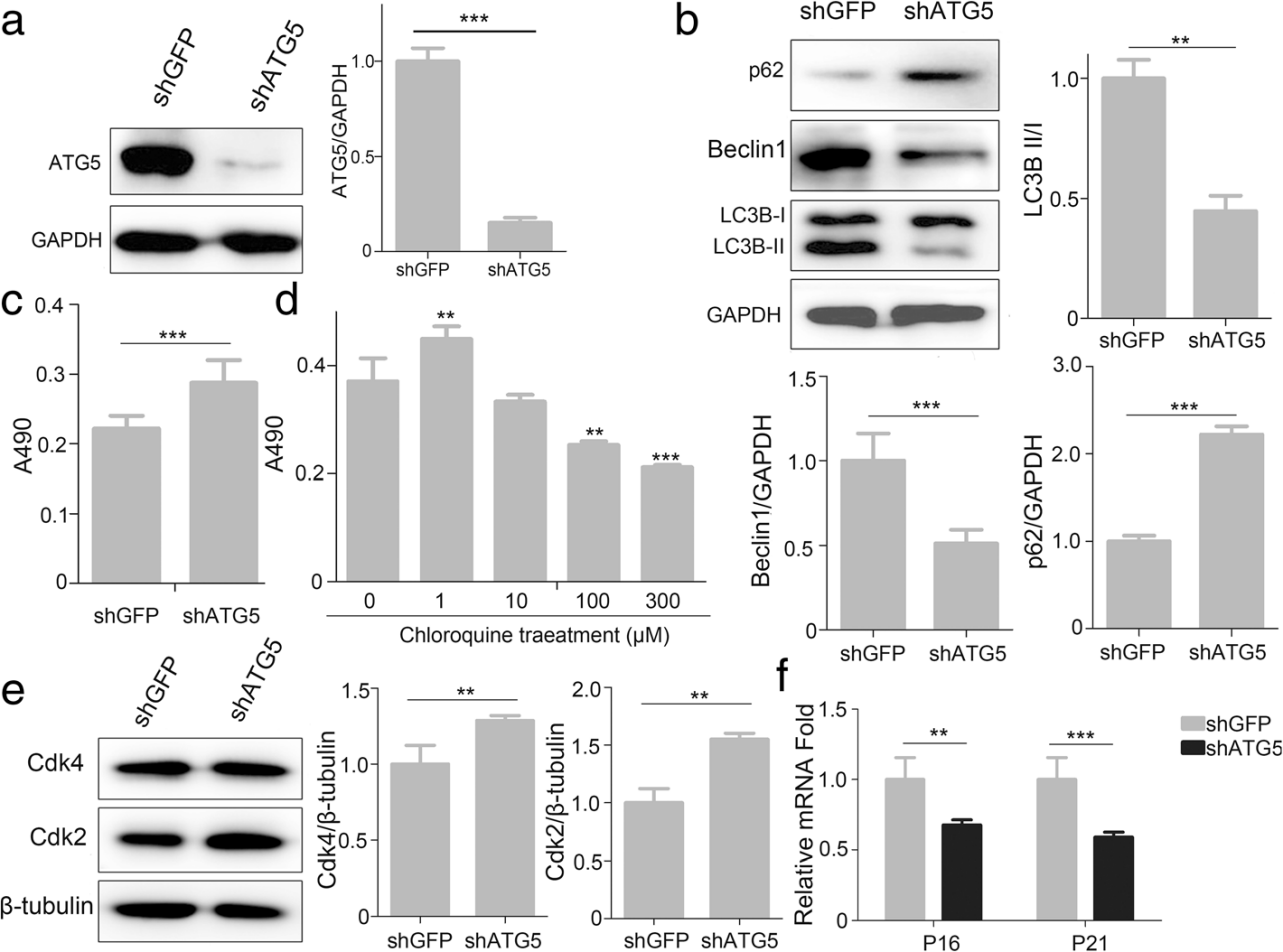
Inhibition of autophagy promotes proliferation of MenSCs. **a** Representative WB of ATG5 in MenSCs infected with lentivirus expressing shGFP or shATG5. *t* test, ***p < 0.001. **b** WB was used to detect autophagy proteins, LC3, Beclin1, and p62 in MenSCs after infected with lentivirus expressing shGFP or shATG5. *t* test, **p < 0.01 and ***p < 0.001. **c** Cell proliferation assay between the shGFP and shATG5 groups using MTT assay. *t* test, ***p < 0.001. **d** Cell proliferation with chloroquine at different concentration was assessed using MTT assay, one-way ANOVA followed by Dunnett’s test, **p < 0.01 and ***p < 0.001, versus 0 μM CQ. **e** WB was used to detect Cdk2 and Cdk4 expression in shGFP or shATG5 MenSCs. *t* test, **p < 0.01. **f** Real-time PCR analysis of P21 and P16 mRNA expression in shGFP or shATG5 MenSCs. *t* test, **p < 0.01 and ***p < 0.001. All data are provided as means ± SEM

ATG5 is a key protein involved in the extension of the phagophoric membrane in the autophagic vesicles. To evaluate whether the defection of autophagy effected on the proliferation of MenSCs, we constructed shRNA to knockdown Atg5 expression (Fig. 4a). The data showed that knockdown of Atg5 depresses autophagic activity effectively through the expression pattern of LC3, p62, and Beclin1 (Fig. 4b). Then, we found that inhibition of autophagy stimulates proliferation of MenSCs by shAtg5 (Fig. 4c). Chloroquine has been evaluated as an autophagy blocker and increases the proliferation of MenSCs at a low concentration (Fig. 4d). Furthermore, we show the protein level of Cdk2 and Cdk4 were increased, and transcript mRNA expression of P16 and P21 were decreased obviously in MenSCs transfected with shAtg5 (Fig. 4e, f). These results indicate that inhibition of autophagy improves the proliferation rate of MenSCs.

### Effects of Gsk3β inhibitor CHIR99021 on the G0/G1 phase arrest induced by excessive autophagy in MenSCs

**Fig. 5:**
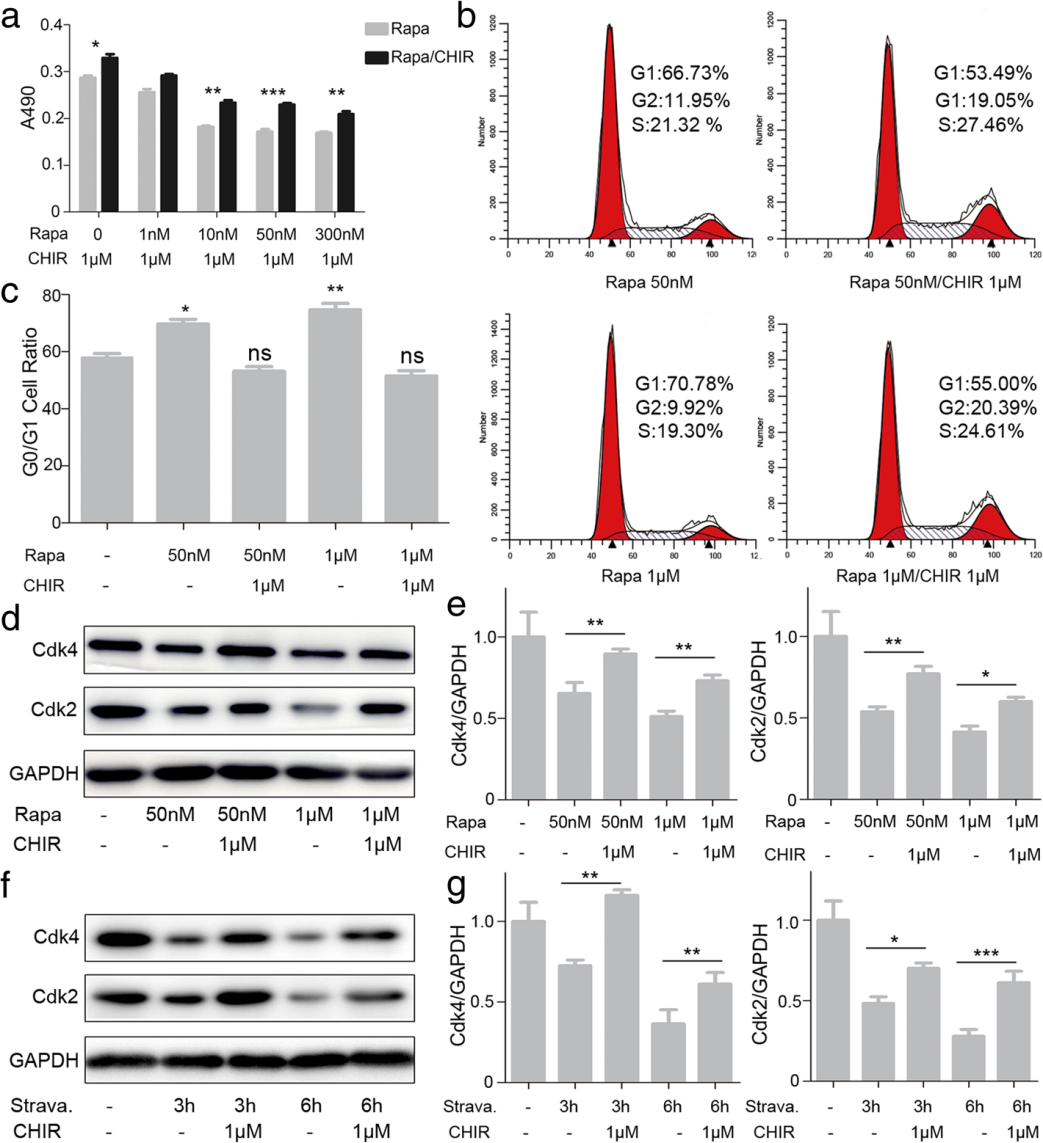
Effect of Gsk3β inhibitor CHIR99021 on autophagy-induced cell cycle arrest of MenSCs. **a** Cell proliferation assay in MenSCs treated with rapamycin or/and CHIR99021, one-way ANOVA followed by Tukey test, *p < 0.05, **p < 0.01, and ***p < 0.001. **b**, **c** Cell cycle distribution was detected by flow cytometry after treatment in both cancer cells, one-way ANOVA followed by Dunnett’s test; ns represents not significant, *p < 0.05 and **p < 0.01, versus left column. **d**–**g** WB was used to detect Cdk2 and Cdk4 expression in MenSCs after treatment in different combinations. One-way ANOVA followed by Tukey test; ns represents not significant, *p < 0.05,**p < 0.01, and ***p < 0.001. All data are provided as means ± SEM

GSK3β, a “destruction complex” [18] and a key element of the Wnt/β-catenin pathway, can promote cell proliferation, differentiation, and growth through the phosphorylation and degradation of β-catenin [19]. CHIR99021 is a potent and high specific inhibitor of GSK3β, which is used to mimic the biochemical effect of Wnt signaling [20]. We further investigated the correlation between CHIR99021 and the G0/G1 phase arrest induced by excessive autophagy. MTT assay was carried out on MenSCs treated with rapamycin (10, 50, and 300 nM and 1 μM) and CHIR99021 (1 μM) for 24 h (Fig. 5a). We found that CHIR99021 can alleviate proliferative inhibition of MenSCs caused by rapamycin. Meanwhile, it was found that CHIR99021 can protect MenSCs against G0/G1 phase arrest caused by rapamycin using PI staining (Fig. 5b, c). Next, the western blotting analysis showed that the inhibitory effects on Cdk2 and Cdk4 expression in rapamycin-treated cells were reversed by Gsk3β inhibitor CHIR99021 (Fig. 5d, e). Further, we performed the same experiment using starvation, which was consistent with the rapamycin results (Fig. 5f, g). Taken together, our data indicates that autophagy induces G0/G1 phase cell cycle arrest in a Gsk3β-dependent manner.

### Autophagy inhibits cell proliferation through the GSK3β/β-catenin pathways in MenSCs

**Fig. 6:**
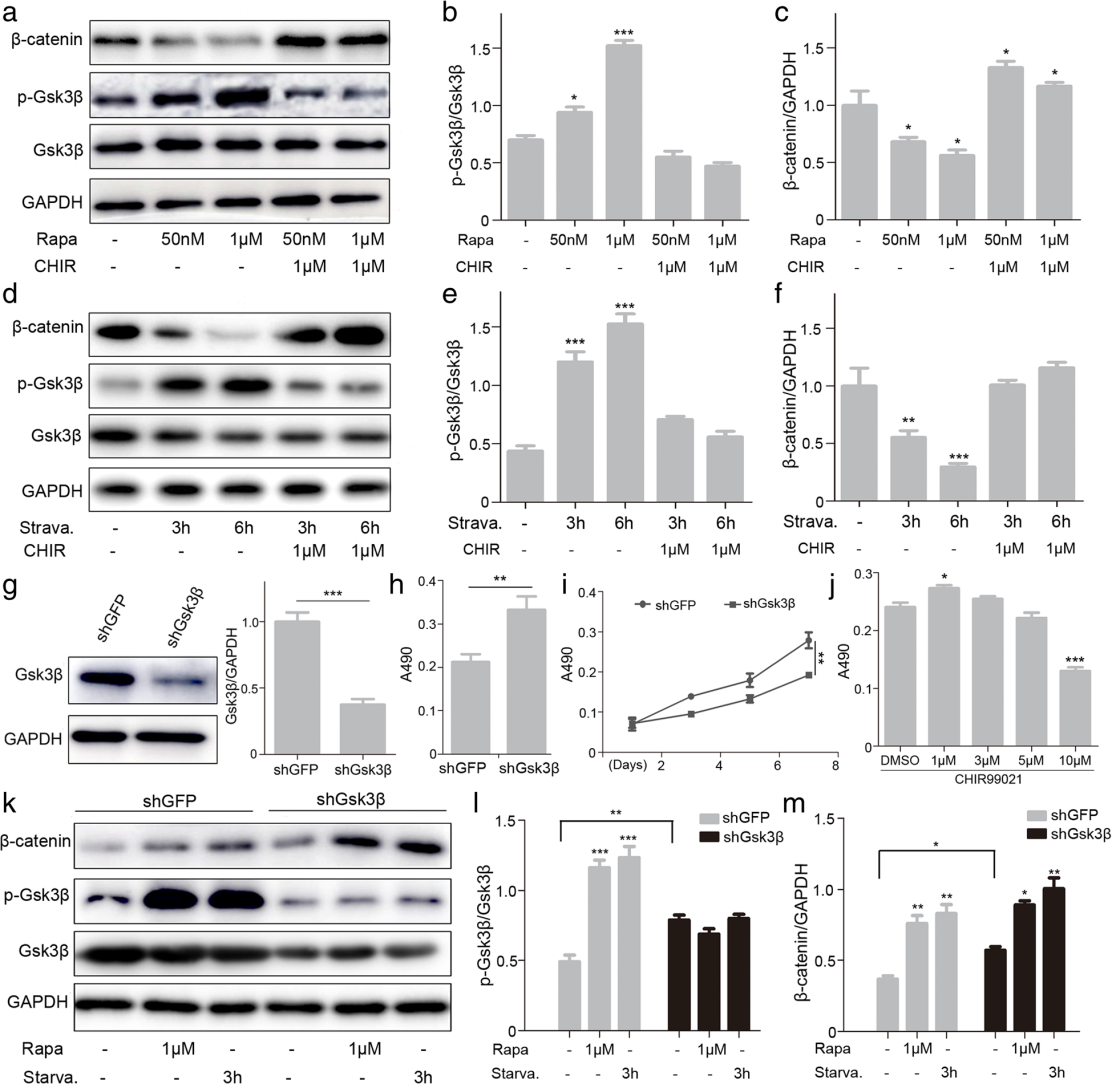
Autophagy inhibits cell proliferation through the GSK3β/β-catenin pathways in MenSCs. **a**–**f** WB was used to detect p-Gsk3β, GSK3β, and β-catenin in MenSCs after treatment in different combinations, one-way ANOVA followed by Dunnett’s test, *p < 0.05, **p < 0.01, and ***p < 0.001, as compared to the left column. **g** WB of GSK3β in MenSCs infected with lentivirus expressing shGFP or shGSK3β. *t* test, ***p < 0.001. **h**, **i** Cell proliferation was assessed between shGFP and shGSK3β using MTT assay. *t* test, **p < 0.01. **j** The proliferation capacity during the 24-day culture was determined by MTT assay in MenSCs treated with CHIR99021, one-way ANOVA followed by Dunnett’s test, *p < 0.05 and ***p < 0.001, versus DMSO. **k**–**m** WB of p-Gsk3β, GSK3β, and β-catenin in shGFP or shGSK3β MenSCs after treatment. Statistical analysis is based on one-way ANOVA followed by Tukey test; ns represents not significant, *p < 0.05,**p < 0.01, and ***p < 0.001. All data are provided as means ± SEM

Since Gsk3β inhibitor CHIR9921 can reverse the G0/G1 phase arrest and proliferative inhibition in MenSCs caused by excessive autophagy, we speculated that GSK3β/β-catenin pathway might play important roles in the inhibitory effect caused by this event. Western blot results showed that the phosphorylation level of Gsk3β was markedly increased, and the β-catenin level was reduced following the incubation of MenSCs with rapamycin for 24 h. However, the changes in protein expression levels of p-Gsk3β and β-catenin caused by overactivated autophagy were reversed by Gsk3β inhibitor CHIR99021 (Fig. 6a–c). We obtained similar experimental results when we handled MenSCs with starvation or/and CHIR99021 (Fig. 6d–f).

To further confirm the important role of Gsk3β in autophagy-induced cell cycle arrest and suppressed cell division, we delivered a shRNA against Gsk3β in MenSCs. Cells infected with lentivirus expressing shGSK3β showed obvious lower levels of GSK3β and much higher multiplication rate compared to shGFP cells (Fig. 6g–i). Meanwhile, the proliferation rates of MenSCs were increased when treated with Gsk3β inhibitor CHIR99021 in a certain concentration range (Fig. 6j). Furthermore, we detected the protein levels of p-Gsk3β and β-catenin after treated with rapamycin or starvation in the shGFP and shGsk3β groups. Compared with shGFP MenSCs, the ratio of Gsk3β phosphorylation obviously has little changes in the shGsk3β group (Fig. 6k–m). The results suggest the phosphorylation level of Gsk3β is more sensitive to autophagy. In summary, our data illustrate the GSK3β/β-catenin pathway may play a key role in the inhibitory effect caused by excessive autophagy.

### Autophagy-induced apoptosis of MenSCs

**Fig. 7:**
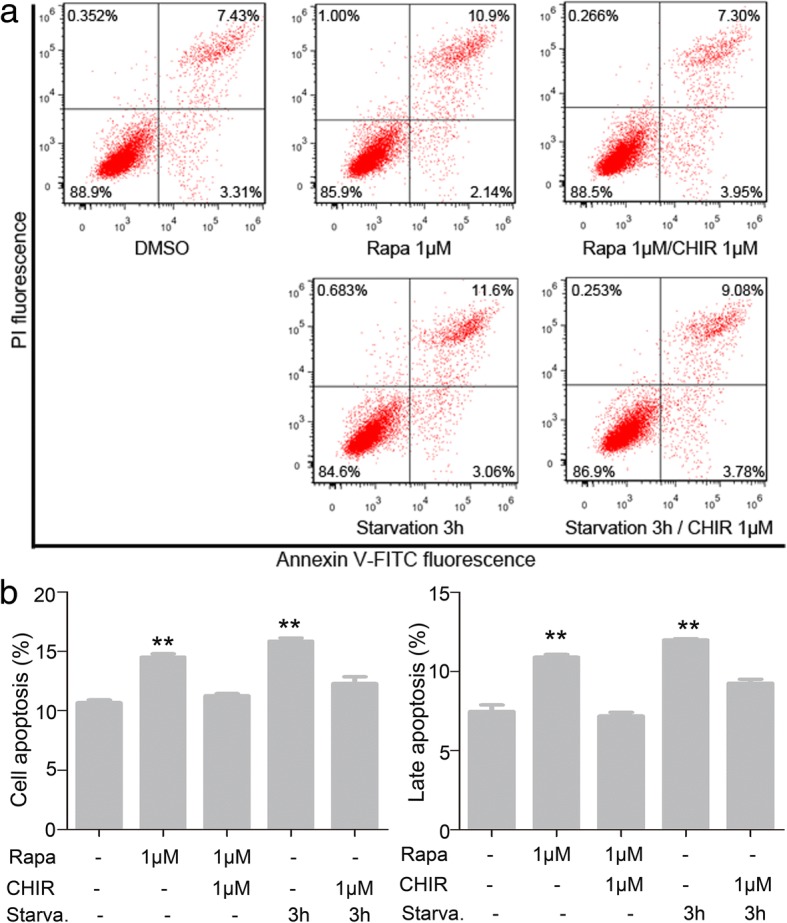
Autophagy-induced apoptosis of MenSCs. **a** Effect of Gsk3β inhibitor CHIR99021 on autophagy induced apoptosis of MenSCs. **b** Statistical analysis of the proportion of apoptotic cells after treatment, one-way ANOVA followed by Dunnett’s test, **p < 0.01, as compared to the left column. All data are provided as means ± SEM

Cell cycle arrest is closely related to cell apoptosis. When the cell cycle checkpoints are abolished, the cells will undergo an apoptotic cascade [21]. Thus, we hypothesized that autophagy might induce apoptosis of MenSCs. Subsequently, Annexin V-FITC/PI double staining was performed using flow cytometry to evaluate the effect of the apoptosis induced by autophagy. The results showed that apoptosis cells increased slightly after treatment with rapamycin or starvation, demonstrating that apoptosis was induced mildly (Fig. 7a, b). In order to analyze the roles of Gsk3β in autophagy-induced apoptosis, cells were treated with CHIR99021 and rapamycin and starvation (Fig. 7a, b). The results showed that CHIR99021 reduces cell apoptosis caused by autophagy in MenSCs.

### Inhibition of Gsk3β enhances MenSCs survival in vivo

**Fig. 8:**
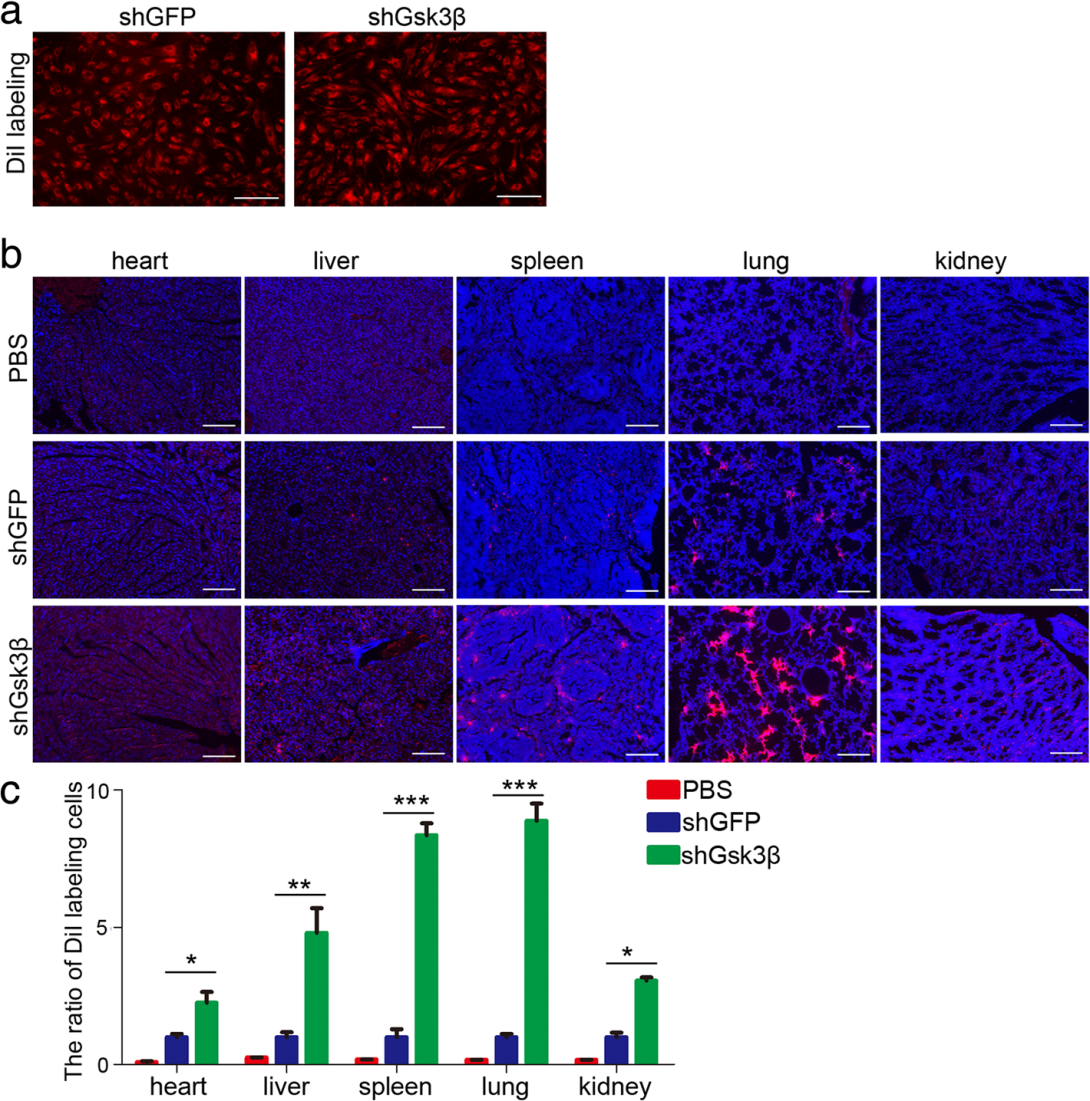
Inhibition of Gsk3β enhances MenSCs survival in vivo. **a** shGFP and shGsk3β MenSCs were labeled with DiI before injection. **b, c** One week later, MenSCs were analysis in the heart, lung, liver, spleen, and kidney among the PBS, shGFP, and shGsk3β groups. *n* = 4 biological replicates, one-way ANOVA followed by Tukey test, *p < 0.05, **p < 0.01, and ***p < 0.001, shGFP versus shGsk3β. Data are provided as means ± SEM. Scale bars, 100 μm

In vivo localization of mesenchymal stem cells after transfer is vital for elaborating the functions of them. To explore the effect of Gsk3β/β-catenin pathway on the MenSCs survival in vivo, we used frozen histological section and image to follow the distribution of MenSCs labeled with DiI after injection into the mouse’s tail vein. Therefore, we injected shGFP and shGsk3β MenSCs into mice intravenously (Fig. 8a). One week later, we analyzed various organs and observed that MenSCs mainly be detained in the heart, lung, liver, kidney, and spleen. The cells are largely distributed in the lung, some in the liver and spleen, and few in the heart and kidney. However, much more DiI-labeled cells were observed in the shGsk3β MenSCs compared to the control group (Fig. 8b, c). Taken together, these results suggest inhibition of Gsk3β function contributes to the survival of MenSCs in vivo.

## Discussion

During the past decade, strong evidence has been demonstrated MSCs-based therapy is a rapidly developing field for diseases due to their multiple modes of action that collectively respond to many of the fundamental pathologic events [22, 23]. MSCs were originally identified in the bone marrow and later detected in many other tissues. Although the benefits of MSCs therapy have been reported for a variety of diseases, some limitations exist when using stem cells for clinical trials. The major hurdles include cell senescence as well as survival and expansion for clinical application.

Lately, experimental findings and clinical trials have focused on the ability of MSCs to migrate to damaged tissue and to produce paracrine factors with anti-inflammatory properties, resulting in the functional recovery of the damaged tissue. MSCs exert their therapeutic potential including paracrine secretion and immunoregulation depending on the survival of stem cells in vivo. However, death induced by environmental conditions and the limited expansion number in vivo are the major obstacles in MSC-based therapy. Therefore, how to prolong MSCs survival is particularly important for the clinical medicine of stem cells.

In recent years, MenSCs have been regarded as a promising stem cell population for cell therapy of different diseases due to their natural high richness and non-invasive sample collection [24]. However, there are few reports about molecular mechanisms and signaling pathways involved in MenSCs biology. Autophagy is a basic cellular homeostatic process that enables cells to dispel partial cytoplasmic contents, and it involves the degradation of cellular components to ensure a cell’s survival [25–27]. Although autophagies induced by rapamycin or starvation are two different processes, they can share a common mechanism. For instance, mTORC1 effectors can respond differentially to the same signals whether rapamycin or starvation treatment [28]. In addition, overexpression of MIR181A resulted in the attenuation of starvation- and rapamycin-induced autophagy [29]. Interestingly enough, autophagy is induced in the endometrial glandular epithelial cells and endometrial stromal cells throughout the menstrual cycle in normal endometrial tissues. Research shows mTOR inhibition promotes endometriotic cell apoptosis via autophagy induction [30]. Moreover, the ability of cells to undergo autophagy is reduced in the ectopic and eutopic endometrium of patients with endometriosis, and autophagy has been shown to be related to the pathogenesis and progression of endometriosis [31]. These studies suggest that there is a relationship between autophagy and endometrial stromal cell survival. Recently, it has been reported that the modulation of autophagy in umbilical cord-derived MSCs may present a novel strategy to improve MSC survival in sepsis and other inflammatory diseases [32]. Meanwhile, inhibition of autophagy significantly improved the therapeutic effects of MSCs on experimental autoimmune encephalomyelitis by regulating the activation and expansion of CD4+ T cells [33]. However, our results illustrate for the first time that autophagy induces G0/G1 arrest and apoptosis in MenSCs via Gsk3β/β-catenin pathway. We demonstrate that overactivation of autophagy in MenSCs by rapamycin and starvation enhances the expression of p-Gsk3β protein which can promote the phosphorylation and degradation of β-catenin.

MSCs express a certain level of the endogenous Wnt protein and regulate their own Wnt/β-catenin signaling to maintain proliferation and function through an autocrine or paracrine loop [34]. It has been demonstrated the association of LiCl concentration with BMSCs proliferation and differentiation. While low concentration of LiCL stimulates BMSCs proliferation, at high concentration levels, LiCl causes a concentration-dependent inhibition of proliferation [35]. One article presents basic information about the role of Wnt signaling in MenSCs proliferation [36]. These results are similar to our data of CHIR99021. Autophagy induced by rapamycin or nutrient deprivation attenuated the expression of Wnt reporter and target genes, while knockdown of LC3 or Beclin-1 potentiated Wnt signaling [37, 38]. However, the relationship of autophagy and Wnt signaling remains unclear in MenSCs. The present study has demonstrated that phosphorylated Gsk3β was upregulated by autophagy, and activation of autophagy inhibited proliferation of MenSCs. Reduced Gsk3β protein could also effect on the survival of MenSCs in vitro and in vivo. This study provided a novel mechanism how autophagy effects on MSCs survival, and inhibited autophagy or Gsk3β can be used to enhance cell survival and maintain long-term stemness in the therapeutic use of stem cells.

## Conclusions

Autophagy is an important factor for the survival of MenSCs. Experimental results show that excessive autophagy induced G0/G1 cell cycle arrest and apoptosis of MenSCs via GSK3β/β-catenin pathway. Further, inhibiting GSK3β levels may improve the survival rate of MenSCs in vivo, thus playing roles in MenSCs therapy.

## Abbreviations

ATG5: Autophagy-related 5
DiI: 1,1′-Dioctadecyl-3,3,3′,3′-tetramethylindocarbocyanine perchlorate
Gsk3β: Glycogen synthase kinase 3β
MenSCs: Menstrual blood-derived endometrial stem cells
MSCs: Mesenchymal stem cells
